# Postoperative remote lung injury and its impact on surgical outcome

**DOI:** 10.1186/s12871-019-0698-6

**Published:** 2019-03-04

**Authors:** Lin Chen, Hailin Zhao, Azeem Alam, Emma Mi, Shiori Eguchi, Shanglong Yao, Daqing Ma

**Affiliations:** 1 Department of Anaesthesiology, Institute of Anaesthesiology and Critical Care Medicine, Union Hospital, Tongji Medical College, Huazhong University of Science and Technology, Wuhan, 430022 Hubei China; 2 Anaesthetics, Pain Medicine and Intensive Care, Department of Surgery and Cancer, Faculty of Medicine, Imperial College London, Chelsea & Westminster Hospital, London, SW10 9NH UK

**Keywords:** Remote lung injury, Cytokine, Pathophysiology, Risk factor, Therapeutic strategy

## Abstract

Postoperative remote lung injury is a complication following various surgeries and is associated with short and long-term mortality and morbidity. The release of proinflammatory cytokines, damage-associated molecular patterns such as high-mobility group box-1, nucleotide-biding oligomerization domain (NOD)-like receptor protein 3 and heat shock protein, and cell death signalling activation, trigger a systemic inflammatory response, which ultimately results in organ injury including lung injury. Except high financial burden, the outcome of patients developing postoperative remote lung injury is often not optimistic. Several risk factors had been classified to predict the occurrence of postoperative remote lung injury, while lung protective ventilation and other strategies may confer protective effect against it. Understanding the pathophysiology of this process will facilitate the design of novel therapeutic strategies and promote better outcomes of surgical patients. This review discusses the cause and pathology underlying postoperative remote lung injury. Risk factors, surgical outcomes and potential preventative/treatment strategies against postoperative remote lung injury are also addressed.

## Background

There are more than 230 million surgical operations around the world each year [1]. Although surgery is an essential treatment pathway in many diseases, respiratory complications following surgery away from the lung, referred to as ‘remote lung injury’, are associated with high mortality and physical disability even 5 years post-surgery [2, 3]. The cost of hospital care associated with such complications is high [4].

Acute lung injury (ALI) and acute respiratory distress syndrome (ARDS) are two common forms of lung injury after surgery. Postoperative remote lung injury affects patient outcomes directly. In this review, the mechanisms of postoperative remote lung injury and its impact on surgical outcomes and prognosis will be discussed. Transfusion is often indispensable in surgery but can have detrimental effects after surgery, including infection and lung injury. According to the Food and Drug Administration, transfusion related acute lung injury (TRALI) is a severe event especially with platelet or plasma containing transfusions among the elderly [5] which is beyond the scope of this review and will not be discussed.

## Epidemiology

A study carried out by Blum et al. demonstrated that the incidence of ARDS in patients who have undergone non-cardiothoracic surgery is 0.2% [6]. Kogan et al. conducted a retrospective study on the incidence and mortality of ARDS in patients after cardiac surgery. Although the incidence is low at just 0.61%, the mortality rate is significantly high at 40.5% [7]. However, others reported that the incidence of ARDS after surgery is as high as 20% and the mortality rate is up to 80% [8]. One recent research indicated that the incidence of ARDS is comparable after abdominal surgery and thoracic surgery with incidences of 3.4 and 4.3% respectively [9]. Although no estimated incidence of ALI has been investigated, a prospective study demonstrated that 1113 out of 6235 patients in the King County Lung Injury Project (KCLIP) cohort suffered from ALI [10]. The observed differences in incidence rate may be due to the different in patient population and protocols used. Interestingly, the incidence of ARDS of all causes in Spain and Northern Europe is lower than that in the United States [11, 12]. Although no comparison between different countries has been discussed, the difference in the incidence of ARDS in various countries may also apply to postoperative ARDS.

## Mechanisms of postoperative remote lung injury

**Fig. 1:**
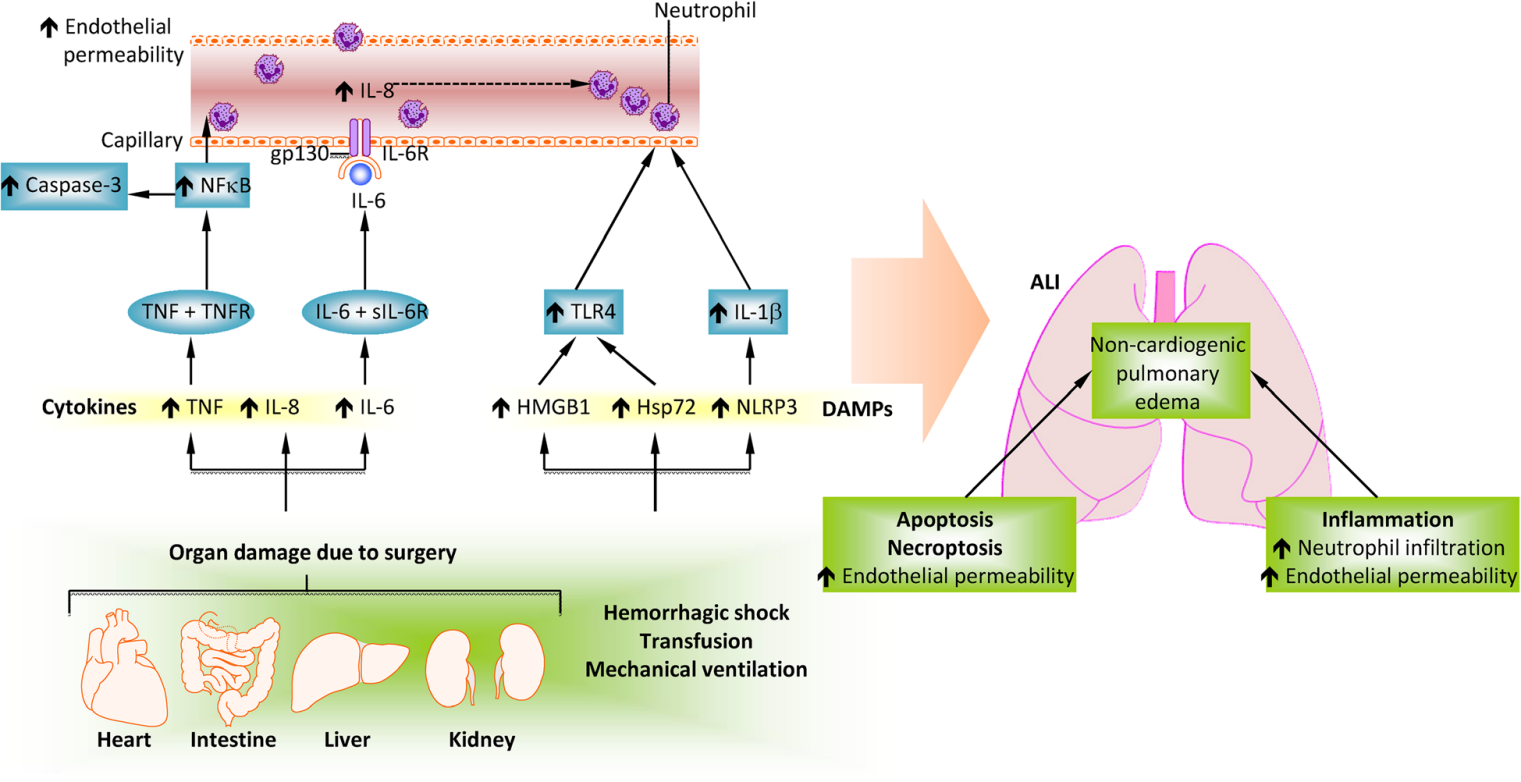
Molecular mechanisms of remote lung injury following other organ injury or disease conditions. Key cytokines involved in lung injury are IL-6, IL-8 and TNF-α, which are induced by acute kidney injury (AKI), cardiopulmonary bypass, renal ischaemia-reperfusion injury, bilateral nephrectomy, transfusion-related acute lung injury and mechanical ventilation. Ischaemic AKI triggers the production of TNF-α which, upon binding to TNFR1, results in NF-κB activation and pulmonary apoptosis. Epithelial cell apoptosis is caspase-3 dependent and can occur following AKI, haemorrhagic shock, sepsis, hepatopulmonary syndrome, acute liver disease and cardiopulmonary bypass, while capillary endothelial cell apoptosis is independent of caspase. Pulmonary epithelial or capillary endothelial cell apoptosis leads to alveolar-capillary barrier dysfunction, causing the accumulation of protein rich fluid in alveoli and subsequent pulmonary oedema. HMGB1 binds to TLR4, leading to the activation of NAD(P) H oxidase in neutrophils, release of ROS, neutrophil infiltration and pulmonary oedema. Derangement of the alveolar capillary barrier causes the release of cytokines and chemokines, facilitating further neutrophil recruitment and the subsequent release of proteases, ROS and cytokines which further damage the barrier and worsen pulmonary oedema (Modified and reproduced with permission) (Springer Nature; Nature Reviews Nephrology) [13]

Remote lung injury often occurs following major surgery due to traumatic injury to primary organs that triggers systemic inflammation [13] (Fig. 1). Pro-inflammatory cytokines and damage-associated molecular patterns (DAMPs) molecules have been shown to play critical roles in mediating detrimental organ cross-talk during the postoperative period.

### Cytokines

Traumatic tissue injury, for example during surgery, causes the release of inflammatory cytokines locally which then spread systemically and subsequently result in organ injury including lung injury. The release of cytokines can be detected in patients after cardiac surgery. Prondzinsky et al. [14] demonstrated that interleukin-6 (IL-6) in plasma was increased in patients after cardiopulmonary bypass (CPB) surgery. Furthermore, the authors found that the correlation between bypass duration and IL-6 is higher in the CPB-coronary artery bypass graft group than in the CPB-percutaneous coronary intervention group, suggesting that surgical trauma contributed to inflammatory response independent of CPB. An increase in the concentration of IL-1β and IL-6 following renal ischaemia-reperfusion injury and bilateral nephrectomy had been noted, whilst IL-6 was shown to play a vital role in causing lung injury after acute renal failure [15, 16]. Vlaar et al. [17] reported that “aged” platelet transfusion induced IL-6 release in animal lung tissue, whilst using a “two-hit” model the authors demonstrated that the supernatant of platelet concentrates were responsible for pulmonary inflammation. It has also been shown that TRALI is associated with both systemic and pulmonary inflammation compared to the control cohorts, as indicated by higher levels of IL-6 and IL-8 in the plasma and bronchoalveolar lavage fluid (BALF) [18, 19].

### DAMPs

DAMPs are endogenous mediators that play critical roles in various diseases. It is thought that DAMPs are transported to the lungs via circulation from organs damaged perioperatively. Although the role of DAMPs in lung injury is not completely understood, several well-described DAMPs are involved in the pathogenesis of postoperative remote lung injury [20, 21].

#### High-mobility group box-1 (HMGB1)

Injured cells and immune cells including macrophages release HMGB1 after surgery, which triggered the production of various pro-inflammatory cytokines. Various surgical procedures, including gastrointestinal surgery and CPB, may induce release of HMGB1 [20, 22]. A study demonstrated that there was an increase in serum HMGB1 after thoracic oesophagectomy, whilst the peak concentration of HMGB1 correlated with length of ICU stay and duration of mechanical ventilation [23]. Moreover, treatment with anti-HMGB1 antibody alleviated lung injury by reducing inflammatory cytokines and inhibiting NF-κB activation [24]. The HMGB1/toll-like receptor 4 (TLR4) signalling pathway had been shown to trigger neutrophil NAD(P) H oxidase activation, which facilitated the release of reactive oxygen species (ROS) [25]. An increase in HMGB1 was also found in ALI induced by liver ischaemia/reperfusion (I/R) injury, whereby binding of HMGB1 to its receptor TLR4 and the subsequent activation of downstream signalling pathways contributed to the development of lung injury [26]. Yamamoto et al. [27] demonstrated that HMGB1 was released into the circulation system after hepatic I/R injury and was related to ALI. Furthermore, they used an HMGB1 absorption column to reduce the concentration of HMGB1 in serum, which attenuated both liver injury and lung injury.

#### NOD-like receptor protein 3 (NLRP3)

NLRP3 is a member of the NLR family that participates in inflammatory response under various cellular stresses. Upon activation, an inflammasome complex is formed which facilitates the maturation and secretion of IL-1β and IL-18, mediated by activated caspase-1 [28, 29]. Moreover, the interaction between extracellular histones and NLRP3 inflammation also mediates ALI [30].

NLRP3 expression is increased in the lung following mechanical ventilation. Kuipers et al. [31] demonstrated that 5 h of mechanical ventilation upregulate NLRP3 mRNA expression in alveolar macrophages from patients. In addition, the increase of NLRP3 mRNA resulted in upregulation of caspase-1 expression and an increase in uric acid level. Compared to the wild-type group, NLRP3 knock-out mice experienced less lung injury after high tidal volume mechanical ventilation. Mechanistic insight was provided by Wu et al., they demonstrated that cyclic stretch activated NLRP3 inflammasome and increased IL-1β production in alveolar macrophages, mediated by mitochondria-generated ROS. Moreover, mechanical ventilation was shown to activate NLRP3 inflammasome in mouse alveolar macrophages and increase IL-1β release in vivo. However, pulmonary inflammatory injury induced by mechanical ventilation was alleviated by IL-1β neutralization [21]. The evidence indicated that macrophage NLRP3 inflammasome may bridge the gap between mechanical stretch and the release of IL-1β. Recently, it was reported that autophagy in alveolar macrophages contributed to the pathogenesis of lung injury during mechanical ventilation through activation of the NLRP3 inflammasome [32].

Haemorrhagic shock is capable of activating NLRP3 inflammasome. In lung endothelial cells, ROS derived from haemorrhagic shock-activated NAD(P) H oxidase induced inflammasome activation and IL-1β secretion. Inflammasome activation is amplified by ROS released by neutrophils which further enhance NAD(P) H oxidase activation [33]. Imbalance of NLRP3 inflammasome activation and its negative-feedback regulator pryin also contributed to ALI following haemorrhagic shock [34].

#### Heat shock proteins (Hsp)

Hsp are another type of DAMPs released by cells under stress and noxious stimuli in a process known as stress protein response (SPR). SPR can be activated by hyperthermia and various environmental insults such as oxidative stress toxins [35]. Increasing evidence indicated that Hsp were involved in the process of lung injury. The presence of extracellular Hsp72 (eHsp72) has been reported in the pulmonary oedema fluid of patients with ALI. In addition, eHsp72 release was found in mice under SPR activation [36]. Chase et al. [37] further validated the relationship between Hsp72 and lung inflammation. Intratracheal instillation of Hsp72 caused TLR-4 dependent cytokine release and neutrophil recruitment in BALF. In vitro, Hsp72 directly activated airway epithelial cells and induced upregulation of IL-8 expression, which was NF-κB dependent. Hsp70 was able to trigger pro-inflammatory signals in macrophages through toll-like receptors [38]. Various studies have reported that in patients after major surgery, there was an increased release of Hsp70 into the circulation [39–41]. Interestingly, circulating Hsp70 after major surgery was associated with an increased expression of IL-6 in plasma, whilst both were found to be involved in postoperative organ dysfunction [40]. However, the concentration of Hsp70 declined immediately after surgical insult, suggesting that it only initiated injurious processes upon interaction with its receptor.

### Apoptosis and necroptosis

Apoptosis in inflammatory cells and alveolar cells mediated ALI after haemorrhagic shock [42]. In an indirect ALI mouse model caused by haemorrhagic shock and sepsis, lung inflammation was found to be characterized by caspase-3 dependent lung epithelial cell apoptosis [43, 44]. In addition, caspase-3 dependent pulmonary injury was evident during the pathogenesis of hepatopulmonary syndrome, whilst lung injury secondary to liver injury could be alleviated with administration of caspase-3 inhibitor Z-DEVD-FMK [45]. Pulmonary injury induced by intestinal I/R was also characterized by increased TUNEL positive cells and caspase-3 activity in the lung [46]. Another study demonstrated that ischaemic acute kidney injury triggered tumor necrosis factor-α (TNF-α) production which, through binding to its receptor TNFR1, resulted in pulmonary apoptosis by activating NF-κB [47]. However, in term of programmed cell death, pulmonary epithelial and endothelial cells react differently. Barlos et al. proved that apoptosis of epithelial cells was caspase-dependent, whilst endothelial cells underwent apoptosis in an apoptosis-inducing factor-dependent, caspase-independent manner [48]. Surgical operations such as cardiopulmonary bypass may also cause pulmonary inflammatory response. Increased activity of caspase-3 in the lung has been noted during this process [49].

Recently, it was reported that regulated necrosis also participates in remote lung injury. Two different forms of regulated necrosis, necroptosis and parthanatos, were found to be present in lung injury after kidney transplantation in rats. TNF-α was responsible for activation of key elements of necroptosis and parthanatos in lung epithelial cells, whilst blocking their function with inhibitors alleviated remote lung injury [50].

## Pathology of postoperative remote lung injury

Postoperative remote lung injury shares the same pathological changes as ALI/ARDS. ARDS is characterized by increased permeability of the alveolar epithelium and capillary endothelium. The disruption of these barriers results in the accumulation of protein-rich fluid in the alveoli, causing pulmonary oedema.

**Fig. 2:**
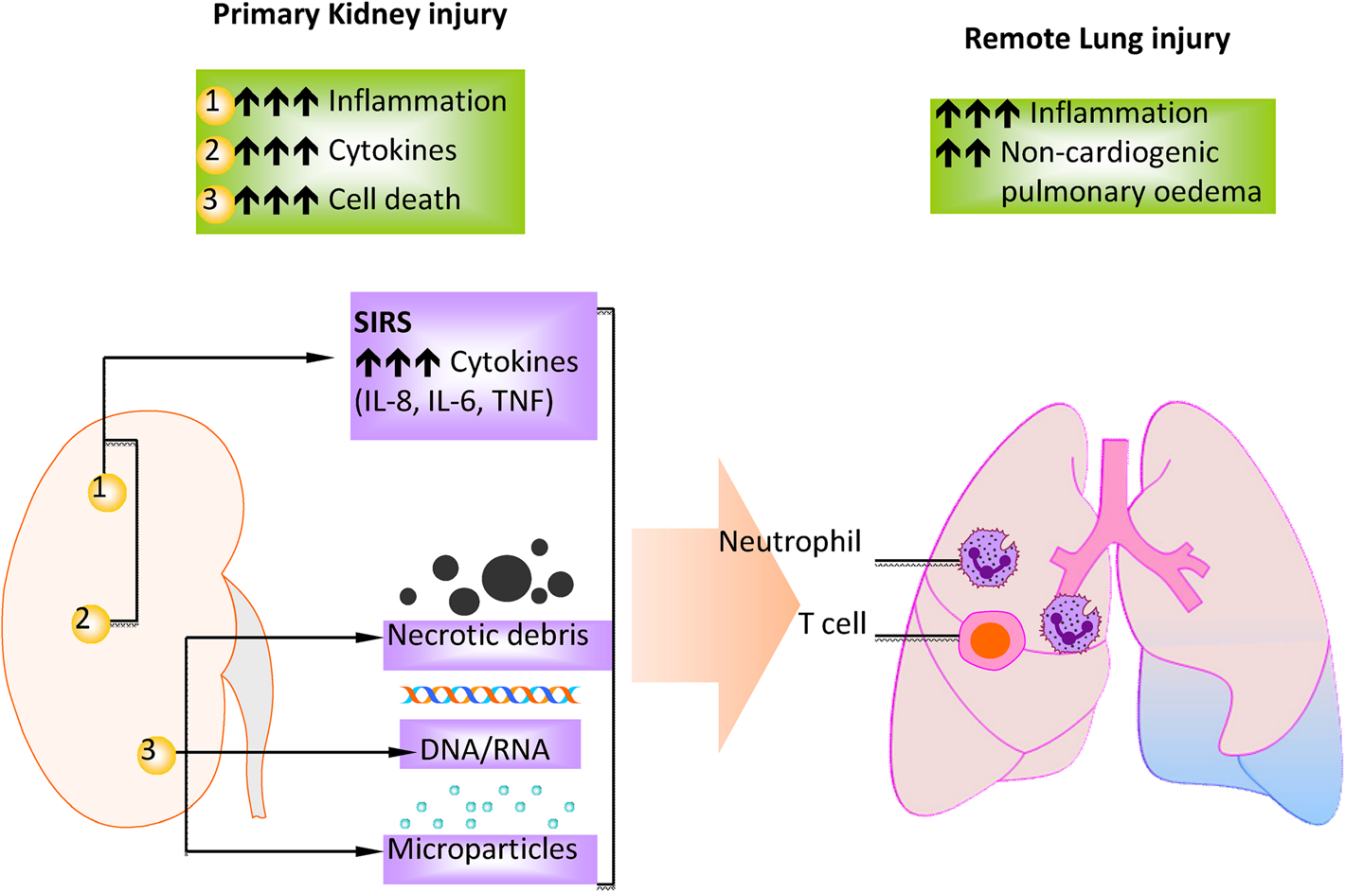
Primary kidney injury and remote lung injury. Primary kidney injury causes the release of DAMP molecules, which in turn results in the upregulation of inflammatory responses in the distant lung. Immune cells, such as neutrophils, monocytes and T cells, contribute to the exacerbation of remote lung injury (Modified and reproduced with permission) (Springer Nature; Nature Reviews Nephrology) [13]

**Fig. 3:**
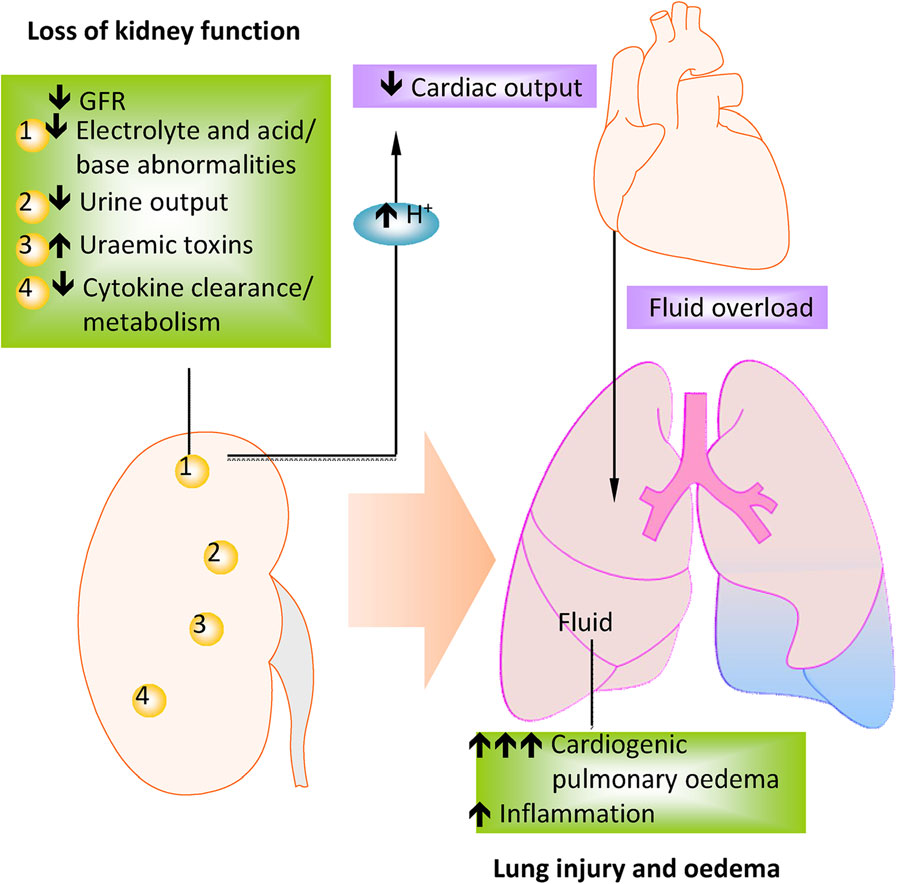
Lung oedema due to functional loss of kidney. Factors associated with the initiation of remote lung injury include the accumulation of toxic by-products, enhanced cytokine release and impaired metabolism due to an imbalance of mediators secreted in kidney injury. These insults cause an increase in pulmonary vascular permeability and, therefore, oedema. Key cytokines in the pathogenesis of remote lung injury following AKI are IL-6 and IL-8, which lead to endothelial dysfunction and pulmonary oedema (Modified and reproduced with permission) (Springer Nature; Nature Reviews Nephrology) [13]

Taking remote lung injury following kidney surgery as an example, kidney injury after surgical operation causes the release of cytokines and chemokines as well as DAMPs which attract a large number of immune cells, such as polymorphonuclear leukocytes (i.e., neutrophils) and T cells into the alveolar space [13] (Fig. 2). Activated macrophages recruit neutrophils and circulating monocytes to the site of injury in the lung. Neutrophils at injured sites release proteases, ROS and other inflammatory mediators, which further damage the barrier, worsening the pulmonary oedema [51]. Moreover, the inflammatory mediators damage type II pneumocytes, which are responsible for producing surfactant. The destruction of these cells results in an increase in surface tension and subsequent collapse of alveoli [52]. Subsequently, the normal structure of lung tissue is disrupted. The injured lung is characterized by thickened alveolar septa, infiltration of neutrophils even red blood cells in lung tissue as well as the accumulation of protein-rich fluid in the alveolar spaces [13, 53] (Fig. 3). The combination of all these processes leads to impairment of gas exchange between the blood stream and alveolar space, resulting in hypoxemia.

## Surgical outcome of patients with postoperative remote lung injury

Overall surgical outcome for patients who have developed lung injury after surgery is not optimistic. Kinugasa et al. [54] demonstrated that postoperative pulmonary complications (PPC) are directly related to an increase in inpatient deaths within ninety days after oesophagectomy. Patients who developed postoperative pneumonia also had a lower five-year overall survival rate compared to those without (26.7% VS 53.4%, hazard ratio = 5.380). Furthermore, pneumonia is one of two independent risk factors to predict poor long-term survival, whilst the other being pathological tumor stage. Another study conducted by Khuri et al. [55] showed that occurrence of pulmonary complications within the first thirty days after major surgery resulted in lower survival rate during the eight-year follow up period.

In a retrospective single centre study conducted in Japan, 502 patients underwent oesophageal carcinoma resection were enrolled and their postoperative complications were analysed [56]. The overall incidence of postoperative complications was 43% with the incidence of PPC was 19.7%. Importantly, patients who developed PPC experienced a decreased overall survival rate with a multivariable hazard ratio of 1.60.

Recently, a meta-analysis discussed the relationship between postoperative lung injury and the incidence of mortality and mobility after abdominal or thoracic surgery [9]. Among 3365 patients, the overall incidence of postoperative lung injury was approximately 4% regardless of types of surgery; however, postoperative lung injury after thoracic surgery was associated with higher patient morbidity. It was found that lung protective ventilation during surgery resulted in a lower incidence of postoperative lung injury, although it has no effect on postoperative mortality. In addition, patients with postoperative lung injury or PPC required longer mechanical ventilation support as well as longer ICU and hospital stay, which contributed towards a higher financial burden on healthcare system [9, 57, 58].

## Predictions and prevention for postoperative remote lung injury

Postoperative remote lung injury and/or PPC are not uncommon following major surgical procedures [57, 59, 60]. As a result, it is important to be able to predict risk factors and consider appropriate precautions. In 1997, Brooks-Brunn analysed data from 400 patients underwent abdominal surgery [61]. Six out of twenty-three risk factors were identified: age over 60, impaired preoperative cognitive function, smoking history within the past 8 weeks, body mass index over 27, history of cancer and the incision-site. In another prospective survey with patients underwent general elective surgery, postoperative nasogastric intubation, preoperative sputum production and longer duration of anaesthesia were identified as modifiable risk factors [60]. Given the high rate of pulmonary complications and the high mortality rates following oesophagectomy, Law et al. [62] analysed data from 421 patients to further investigate potential predictive factors for PPC. Logistic regression analysis identified three predictive factors for PPC: advanced age, operation period and proximal tumor location.

In 2010, a large prospective, multicentre study reported that 5% of patients developed PPC [1]. Seven independent risk factors were identified, including low preoperative arterial oxygen saturation, acute respiratory infection in the last month, age, preoperative anaemia, upper abdominal or intrathoracic surgery, surgical operation lasting more than 2 h and emergency surgery. In another multicentre cohort research, Kor et al. [63] re-evaluated the risk factors for post-surgical ARDS. In contrast to the previous study in 2010, they found nine independent risk factors contributed to postoperative lung injury: sepsis, high-risk aortic vascular surgery, high-risk cardiac surgery, emergency surgery, cirrhosis, admission locations other than home, increased respiratory rate, FIO_2_ greater than 35% and SpO_2_ less than 95%. This study also indicated that the surgical lung injury prediction (SLIP) score is of inferior power in this high-risk population than SLIP-2, which is more accurate in diverse and acutely ill patients. However, due to variation in clinical settings in different centres, there is still no standardized prediction scale available worldwide. Furthermore, one recent study reported by Fernandez-Bustamante et al. showed that PPCs are common in patients in spite of protective ventilation settings [64]. They also revealed 3 non-modifiable risk factors as emergency, surgical site and age, and 5 modifiable factors of colloid administration, preoperative oxygenation, blood loss, anaesthesia duration and tidal volume. All those risk factors are summarized in the Table 1.

**Table 1:** The modifiable and non-modifiable factors in prediction of postoperative remote lung injury

	Modifiable factors	Non-modifiable factors
Preoperative variables	Anaemia FIO_2_ greater than 35% Impaired cognitive function Increased respiratory rate Low arterial oxygen saturation SpO_2_ less than 95% Sputum production	Acute respiratory infection in the last month Advanced age Admission locations other than home BMI over 27 Cirrhosis History of cancer Sepsis Smoking history within the past 8 weeks
Intraoperative varibles	Anaesthesia duration Blood loss Colloid administration Operation period Tidal volume	Emergency surgery High-risk aortic vascular surgery High-risk cardiac surgery Proximal tumor location Surgical site Upper abdominal or intrathoracic surgery
Postoperative variables	Nasogastric intubation	

*BMI* body mass index, *FIO*_*2*_ fraction of inspiration O_2_, *SpO*_*2*_ peripheral capillary oxygen saturation

Identification of risk factors allows clinicians to predict postoperative remote lung injury. However, efforts to improve ventilation strategies during surgery are also vital. Amongst patients without ARDS at the onset of ventilation, fewer patients develop lung injury under protective ventilation compared to conventional ventilation [65]. In a population underwent major abdominal surgery, lung protective ventilation during surgery was associated with a lower incidence of PPC and better clinical outcome [66]. Furthermore, Severgnini et al. [67] reported that protective ventilation during abdominal surgery correlated with improved pulmonary function after surgery. A meta-analysis by Serpa Neto et al. [68] reported that patients ventilated with low tidal volume (VT) are less likely to develop PPC. While another large RCT compared the effect of high or low positive end expiratory pressure (PEEP) on PPC occurrence [69], surprisingly, the strategized combination of high PEEP and recruitment manoeuvres failed to protect against PPC. The authors, therefore, suggested that intraoperative protective ventilation should consist of a low VT and low PEEP without recruitment manoeuvres. Patients may respond to the same ventilation strategy and the same presumed protective ventilation strategy (low VT with high PEEP) may produce controversial results. Different from the result from the PROVHILO trial [68], Spadaro et al. showed that low VT together with PEEP at 10 cm H_2_O is protective during one lung ventilation [70].

## Therapeutic strategies

In recent years there has been an increasing understanding of the possible pathophysiological processes underlying the development of postoperative remote lung injury, with evidence to suggest that it may be possible to exploit this knowledge to reduce its incidence within the clinical environment.

In vivo models of ARDS have demonstrated that various anaesthetic agents, including isoflurane, sevoflurane and desflurane, possess anti-inflammatory and cytoprotective effects [71–73]. These data suggest that volatile anaesthetic agents may possess significant protective effects in ameliorating ARDS as a result of a variety of pathogenic insults. Whilst there is limited clinical evidence specifically purporting the protective effects of these agents against postoperative remote lung injury, given the fact that the various insults studied share common pathogenic pathways with remote lung injury, it is reasonable to ascertain that these volatile anaesthetic agents may too be protective against remote lung injury.

Isoflurane is a commonly used volatile anaesthetic agent [74] and has been shown to possess both anti-inflammatory [75] and cytoprotective [76] properties. Animal models of lung injury, including mechanical ventilation induced lung injury and inhaled endotoxin [71, 77], have demonstrated the potential utility of isoflurane as a pulmonary protectant. Proposed mechanisms include the downregulation of NF-κB by reducing its expression and simultaneously upregulating I-κB expression, whilst also mediating the expression of apoptotic markers, including Bcl-2 and Bax [78, 79], as well as a reduction in vascular leak [71]. Furthermore, isoflurane also attenuated LPS-induced lung injury by inhibiting NLRP3 inflammasome activation [80]. The fact that isoflurane attenuates the activation of common inflammatory pathways suggests that the perioperative attenuation of these pro-inflammatory mediators in patients undergoing surgery may reduce the incidence of remote lung injury.

Sevoflurane, another commonly used inhaled anaesthetic agent, has similarly been demonstrated the ability to ameliorate lung injury in vivo. In animal models of lung injury, sevoflurane has consistently demonstrated its protective properties by reducing deleterious histological changes, reducing wet to dry ratio and improving ventilation parameters [81–83]. Furthermore, sevoflurane administration caused a reduction in neutrophil infiltration, pro-inflammatory cytokine release as well as a reduction in NF-κB expression [84, 85]. The release of inflammatory cytokines has been shown to be involved in the pathogenesis of remote lung injury, once against suggesting that the use of sevoflurane may similarly reduce the incidence of remote pulmonary insults following surgery.

Propofol has been shown to reduce the expression of a similar cytokine profile to sevoflurane, thus conferring a 2-fold increase in survival in an LPS model of lung injury by reducing pulmonary oedema and histological damage via its anti-inflammatory and anti-oxidative properties [86, 87]. Dexmedetomidine (Dex) is a sedative that has been demonstrated anti-inflammatory effects and the ability to ameliorate lung injury. Dex is a potent and highly selective α2-adrenergic agonist that exhibits sedative, analgesic, amnestic, and sympatholytic properties. Previous studies have demonstrated that Dex was able to protect against lung injury following kidney ischaemia-reperfusion injury due to its ant-inflammatory effects [88] and ability to reduce pulmonary microvascular hyper-permeability [89]. Furthermore, Dex reduced caspase 3 and Bax expression, whilst upregulated Bcl-2 expression. As animal studies have indicated the significant role of caspase 3 dependent lung epithelial cell apoptosis in the pathogenesis of remote lung injury [42, 43], including hepatopulmonary syndrome, Dex’s anti-inflammatory effects may reduce the incidence of post-operative remote lung injury in clinical practice but warrant further study.

Another therapeutic approach may be the use of xenon, a novel general anaesthetic agent, that has demonstrated anti-inflammatory and anti-apoptotic effects in models of acute lung injury following renal injury [90].

Furthermore, numerous observational studies have attributed the judicious use of intravenous fluid in the perioperative period with a higher incidence of acute lung injury [91, 92]. One randomized clinical trial conducted by Volta CA et al. showed that patients received balanced solutions experienced lower concentration of active matrix metalloproteinase-9 and higher level of tissue inhibitor of metalloproteinase-1 and IL-10 compared to the unbalanced solutions [93]. Whilst a large RCT of patients with acute lung injury found that conservative administration of intravenous fluids is associated with an improvement in oxygenation, lung compliance and 60-day survival [94]. This is a potentially simple way of reducing the incidence of perioperative lung injury, however must be balanced with the risks associated with dehydration.

## Remaining questions and directions for future research

Whilst the scope for improvement in the incidence of postoperative remote lung injury is significant, there are a number of important unanswered questions. Currently, the vast majority of evidence underlying our current understanding of the pathogenesis of remote lung injury is through in vivo studies, with limited human studies.

The off-license use of anaesthetic agents to ameliorate remote lung injury is inherently associated with risks in terms of safety and efficacy. Only a few human studies exist investigating the therapeutic effects of sevoflurane and propofol in ameliorating postoperative lung injury, with no overall significant identified differences in the incidence of ARDS, as well as conflicting biochemical changes reported between the studies [95–97]. However, historical evidence exists to suggest that inhaled agents, such as isoflurane, may possess deleterious effects [98].

This highlights the importance of high-powered, controlled, clinical trials investigating previously purported in vivo markers of remote lung injury, such as IL-6, HMGB-1 and NF-κB, in human subjects. The aim of this should be to facilitate similarly high-powered, randomised controlled trials investigating the efficacy, safety and side-effect profiles of anaesthetic agents such as isoflurane, sevoflurane and dexmedetomidine within clinical practice, as well as conservative approaches such as non-judicious intravenous fluid administration. Furthermore, additional in vitro and in vivo research is required to investigate the development of novel agents targeting specific pro-inflammatory markers, such as HMGB-1, NLRP3 and Hsp, as these agents may confer additional therapeutic benefit in the future.

## Conclusion

Postoperative remote lung injury is not an uncommon condition clinically, and it is associated with adverse effects on overall patient survival. The pathological mechanisms are complex, involving cytokines and DAMPs released by injured organs or surgical sites. As a result, the normal function of the respiratory system is compromised, contributing to the development of lung injury. Due to the poor outcome associated with postoperative remote lung injury, predicting the population at risk is critical in order to facilitate lung injury prevention at the incipient stage. Approaches to ameliorate the pathological effects of postoperative remote lung injury are still required and necessitate further investigation, particularly utilizing well-powered, randomized controlled trials.

## Abbreviations

ALI: Acute lung injury
ARDS: Acute respiratory distress syndrome
BALF: Bronchoalveolar lavage fluid
CPB: Cardiopulmonary bypass
DAMPs: Damage-associated molecular patterns
HMGB1: High-mobility group box-1
HSP: Heat shock protein
I/R: Ischaemia/reperfusion
IL: Interleukin
NLRP3: NOD-like receptor protein 3
PEEP: Positive end expiratory pressure
PPC: Postoperative pulmonary complication
ROS: Reactive oxygen species
SLIP: Surgical lung injury prediction
SPR: Stress protein response
TLR4: Toll-like receptor 4
TNF-α: Tumor necrosis factor-α
TRALI: Transfusion related acute lung injury
VT: Tidal volume
